# A drug identification model developed using deep learning technologies: experience of a medical center in Taiwan

**DOI:** 10.1186/s12913-020-05166-w

**Published:** 2020-04-15

**Authors:** Hsien-Wei Ting, Sheng-Luen Chung, Chih-Fang Chen, Hsin-Yi Chiu, Yow-Wen Hsieh

**Affiliations:** 1 Department of Neurosurgery, Taipei Hospital, Ministry of Health and Welfare, New Taipei City, Taiwan; 2 Graduate Program in Biomedical Informatics, Yuan Ze University, Taoyuan City, Taiwan; 3 Department of Electrical Engineering, National Taiwan University of Science and Technology, Taipei City, Taiwan; 4 Pharmaceutical Department, Mackay Memorial Hospital, No. 92, Sec. 2, Zhongshan N. Rd, Taipei City, 10449 Taiwan; 5 Department of Pharmacy, China Medical University Hospital, Taichung, Taiwan

**Keywords:** Deep learning, Drug identification, Look-alike and sound-alike (lasa), Medication error, Patient safety

## Abstract

**Background:**

Issuing of correct prescriptions is a foundation of patient safety. Medication errors represent one of the most important problems in health care, with ‘look-alike and sound-alike’ (LASA) being the lead error. Existing solutions to prevent LASA still have their limitations. Deep learning techniques have revolutionized identification classifiers in many fields. In search of better image-based solutions for blister package identification problem, this study using a baseline deep learning drug identification (DLDI) aims to understand how identification confusion of look-alike images by human occurs through the cognitive counterpart of deep learning solutions and thereof to suggest further solutions to approach them.

**Methods:**

We collected images of 250 types of blister-packaged drug from the Out-Patient Department (OPD) of a medical center for identification. The deep learning framework of You Only Look Once (YOLO) was adopted for implementation of the proposed deep learning. The commonly-used F1 score, defined by precision and recall for large numbers of identification tests, was used as the performance criterion. This study trained and compared the proposed models based on images of either the front-side or back-side of blister-packaged drugs.

**Results:**

Our results showed that the total training time for the front-side model and back-side model was 5 h 34 min and 7 h 42 min, respectively. The F1 score of the back-side model (95.99%) was better than that of the front-side model (93.72%).

**Conclusions:**

In conclusion, this study constructed a deep learning-based model for blister-packaged drug identification, with an accuracy greater than 90%. This model outperformed identification using conventional computer vision solutions, and could assist pharmacists in identifying drugs while preventing medication errors caused by look-alike blister packages. By integration into existing prescription systems in hospitals, the results of this study indicated that using this model, drugs dispensed could be verified in order to achieve automated prescription and dispensing.

## Background

Issuing of correct prescriptions is the mainstay of patient safety. Medication errors are the most important problem that influences safety in health care [1]. The most common medication errors are caused by human factors, such as fatigue and inadequate knowledge [2]. In particular, look-alike and sound-alike (LASA) is the lead error at the level of pharmacists or physicians. A good policy to prevent LASA is to change drug names and their packaging [3]. Researchers used chart reviews and mathematical methods to identify problematic pairs of drug names, and constructed an automated detection system to detect and prevent LASA errors [4]. Unfortunately, major problems remain in drug identification: many drugs look alike; drugs are relatively small in size; and a large number of drugs need to be identified. Existing identification solutions still have their limitations [5–7].

However, some assistive tools do exist. Automated dispensing cabinets (ADCs) represent a solution that dispenses drugs automatically [8, 9], and there are many ADC technologies in existence. Some studies have used barcoding for drug identification and prevention of medication errors [9]. Devices that employ radio-frequency identification (RFID) and Bluetooth to identify the positions of drugs have been designed [8]. Most large hospitals use robots; however, there are fewer robots than needed in hospitals with fewer than 100 beds [10]. Other major problems with the use of ADCs are the development of suitable software that can identify drugs accurately without the need for pre-processing of drugs or a large space in the pharmaceutical department before applying the systems. In addition, it needs to be ensured that these systems will not increase the burden on pharmacists during the prescription process [11, 12].

Alternatively, image-based solutions have been developed. Traditional image recognition finds features through algorithms and then classifies images using certain classifiers [13, 14]. Lee et al. encoded color and shape into a three-dimensional histogram and geometric matrix, and encoded the imprint as a feature vector through a Scale Invariant Feature Transform (SIFT) descriptor and a Multi-scale Local Binary Pattern (MLBP) [15]. Taran et al. [16] proposed the use of a variety of traditional artificial feature integration methods to extract high-dimensional drug features from images to achieve identification of blister packages. Saitoh [17] used the local feature and nearest-neighbor search method to sort images of blister packages in a database according to input test images, and sorted blister packages with the most similar shapes and colors through voting scores.

Most significantly, thanks to the vigorous development of Graphics Processing Units (GPUs) for parallel computing, a current mainstream process is to adopt deep learning methods to replace traditional classifiers. Examples include biomedical imaging and wave recognition [18, 19]; speech recognition [20, 21]; biomedical signal detection [18, 19, 22]; cancer identification [19, 22, 23]; potential drug discovery [24, 25]; and adverse drug effects [26]. Images of the drug are pre-processed to obtain the correct viewing angle and drug separation, and the characteristics of the pills are established manually [27]. Drug identification is implemented in a framework based on a Deep Convolutional Network (DCN), and achieved good recognition. In addition, another method of pill identification first finds the location and area of ​​the drug by detecting the edge contour of the pill [27]; then, through a variety of data augmentation methods such as color shift, size adjustment, Gaussian blur, etc., more training samples are generated to solve the problem of sparse training samples. Three GoogLeNet deep learning networks are used as the main classifiers to train the color, shape and characteristics of the pills, and the recognition results of the three models are then combined to obtain the final recognition results [28].

This study focused on the problem of drug identification using visual images of blister packages. We constructed a Deep Learning Drug Identification (DLDI) model that identifies drugs automatically and can assist pharmacists in dispensing prescriptions correctly. Our goal was to illustrate how ‘look-alike’ error can be captured and explained by a convolution-based deep learning network whose working mechanism is in much similarity to the human visionary recognition capability. Subsequently, appropriate solution to extract more detailed nuance differences can be utilized in distinguishing look-alike objects.

## Methods

To investigate how a deep learning network identifies object types, a dataset containing images both sides of 250 types of blister packages were collected for training and testing data of a deep learning network. Identification results in terms of precision, recall, and the combined F1-score were computed, where an identification error can be regarded as an error due to look-alike cases.

### Data resources

**Fig. 1:**
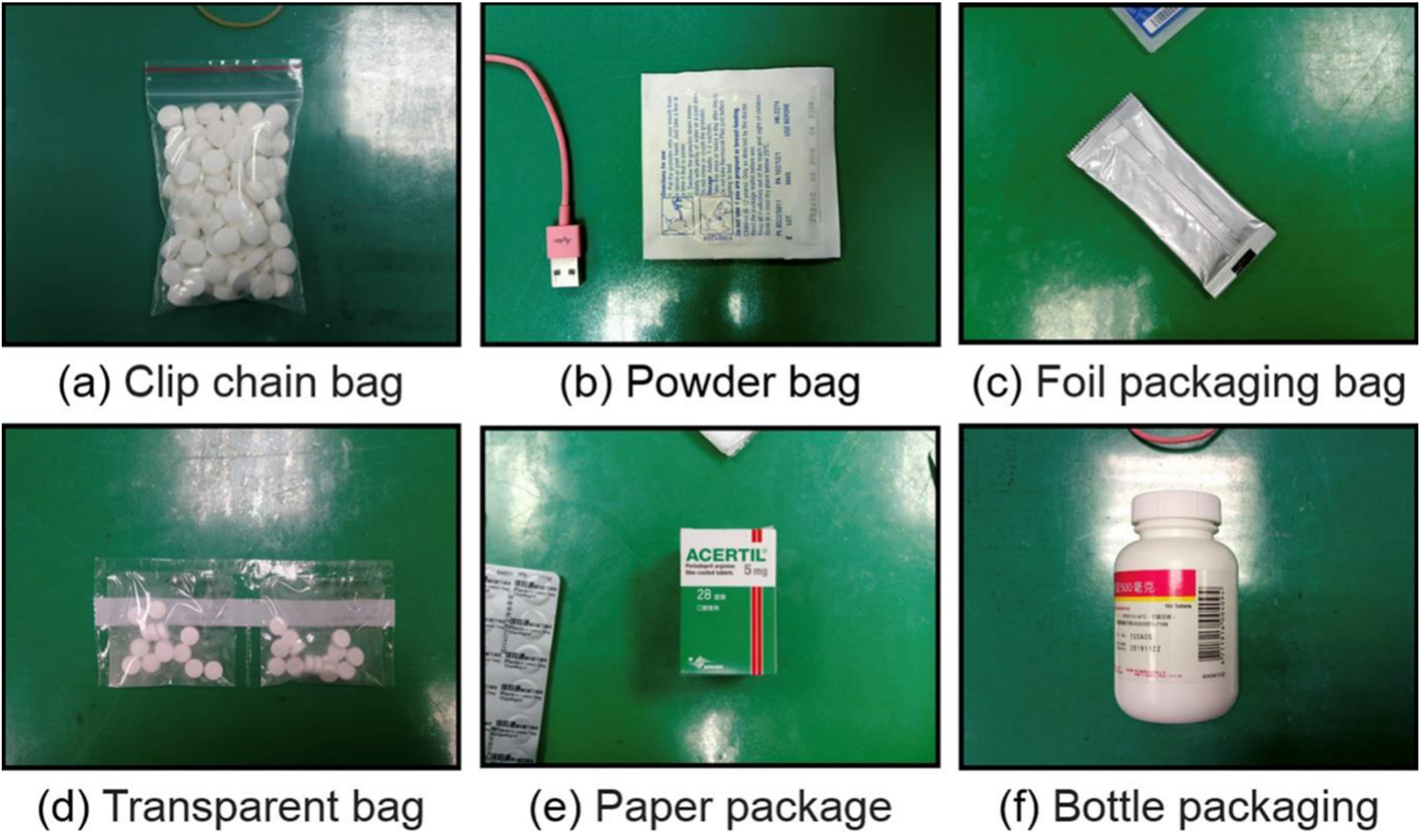
Excluded drug packages. Six classes of drug packaging were excluded: clip chain bags, powder bags, foil packaging bags, transparent bags, paper packages, and bottle packaging

This study collected drugs from the Out-Patient Department (OPD) of a medical center. Of the 272 kinds of drug, this study focused only on recognition of pharmaceutical blister packages. As such, 6 classes of drug packaging (Fig. 1), totaling 32 kinds of drug, were excluded, as follows: clip chain bags, powder bags, foil packaging bags, transparent bags, paper packages, and bottle packaging. The remaining 250 drugs with blister packaging were considered.

**Fig. 2:**
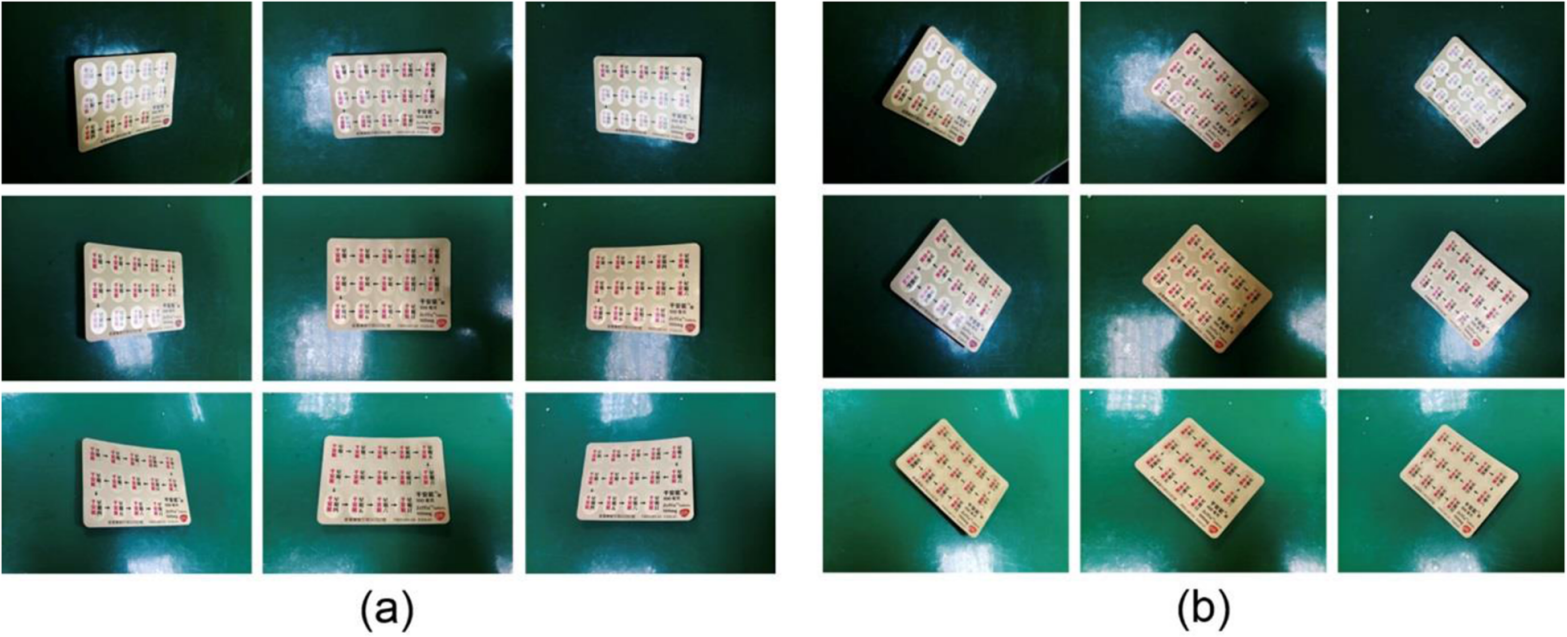
Photographs from different angles**.** Different angles were employed for the camera to focus on, with different rotation directions of the drug packaging. Both front-side and back-side images were obtained for each type of drug

We aimed to identify blister packages by their images, photographed using a camera from different angles. In collecting the training set, 72 images were taken for each side of each type of drug: the camera focused from 9 different angles, with 8 different rotation directions of the drug shown in the images (Fig. 2). Both front-side and back-side images were taken for each drug, resulting in a total of 36,000 images as the training data for deep learning. Images of the front sides of packages contained the shapes and colors of the pills or tablets, whereas images of the back sides contained mostly texture patterns of the drugs or logos of pharmaceutical companies. These images were used to train CNN networks, the deep learning networks, for object identification.

### Deep learning architecture

The concept of the Convolution Neural Network (CNN) was proposed by LeCun and others in 1989. These deep learning networks usually consist of convolutional layers, pooling layers and fully-connected layers [29]. As the convolutional layers and the pooling layers in the network architecture enhance the relationship between pattern recognition and adjacent data, a CNN can be applied to signal types such as images and sounds. Through multi-layer convolution and pooling, the extracted features are treated as inputs, and then forwarded to one or more fully-connected layers for classification. Unfortunately, the simple CNN is not effective for more complex images. Krizhevsky et al. [30] reconstructed a CNN in 2012, and in CNN-based networks, the deep learning framework of “object detection” has also been continuously improved. R-CNN was the first successful CNN-based object detection method, but the speed of detection was very slow [31]. Later, the Fast and Faster R-CNN were constructed [32], optimized on the basis of R-CNN, and the speed and accuracy were improved significantly.

### Software and hardware devices

This study used You Only Look Once (the abbreviation ‘YOLO’ having been proposed by Redmon et al. in 2015) as the solution framework for deep learning [33]. An end-to-end structure was adopted, and compared with the general deep learning method, YOLO focuses on both the area prediction part of detection and the category prediction part for classification. YOLO integrates detection and classification into the same neural network model, with fast and accurate target detection and recognition. These deep learning techniques employ the following features: batch normalization for faster convergence; passthrough for the features identification increasing; hi-res classifier to increase the resolution of the images; direct location prediction to strengthen the stabilization of position prediction; and multi-scale training to improve both speed and accuracy. The SENet and ResNet experiments in this study used the Kubuntu 14.04 system and the Darknet framework in the Caffe structure of Windows 7, which is a special hardware device host for deep learning. This study also employed an Intel® I7–6770 Eight-Core Processor (CPU), 16 GB RAM, and a NVIDIA GTX 1080 Graphic Processing Unit (GPU).

### Experimental design

For model evaluation, this study partitioned the collected data into separate training and testing sets. The training set trained the deep network to generate models, while the testing set evaluated the performance of the constructed models. We randomly choose three-quarters of the 72 pictures of each type of drug for inclusion in the training set, and the remaining quarter were included in the testing set, with 13,500 images in total in the training set and 4500 images in the testing set. This study trained 100 models for each of the front-side and back-side images using the training set. The best model was chosen, which was defined as the model with the greatest accuracy (highest F1 measure) and the fastest speed (fewest Epochs). This study also standardized the YOLO v2 protocol for both the training and testing datasets in each model. All images were converted into 224 × 224 pixels. Neither data augmentation nor pre-training of the model were performed during training. The batch size was 8, meaning that parameters were re-adjusted every 8 images. The highest training frequency was 100 Epochs, one Epoch meaning that the deep network ran all the pictures during training. The parameters were saved after every Epoch was completed (Table 1).

**Table 1:** Training and testing rules of the deep learning network

Size of input image	Adjusted to 224 × 224 pixels
Network built-in data augmentation function	disabled
Pre-trained model	no
Batch size	8
Highest number of training Epochs	100 Epochs (168,800 iterations)
Training weight file storage timing	1 Epoch (1688 iterations)

### Outcome measurement

Confusion matrixes were used to record the results if blister packages were identified, correctly or not. Correct matches were listed on the diagonal of the matrix, whereas cases of missed identification were marked by non-zero values off the diagonal. The higher the number, the greater the chance of misidentification of blister packages of drugs. For example, assume that there is a system for classifying three different drugs (Table 3). Suppose that there are 28 drugs in total: 9 drug A, 6 drug B, and 13 drug C. In this confusion matrix, there are actually nine drug A, but three of them are misidentified as drug B. For drug B, one of the drugs is misidentified as drug C, and two are misidentified as drug A. The confusion matrix shows that it is more difficult to distinguish between drug A and drug B, but easier to distinguish drug C from the other drugs.

The data presented in Table 2 are for the model obtained from 100-Epoch training. The training time, number of training Epochs, precision, recall, and F1 measure were recorded as the evaluation results. The best recognition performance was identified according to the F1 score, and the Epoch number was used to identify the fewest numbers of training Epochs. The recall, also called the true positive rate or the sensitivity, measures the proportion of positives correctly identified. Recall = True Positive / (True Positive + False Negative), of which True Positive denotes a correct identification; while False Negative denotes a misidentified result by taking the correct target as something else. The precision, also called the positive predictive value, measures the proportion of positives among all identified. Precision = True Positive / (True Positive + False Positive), of which False Positive denote a misidentified result by taking something else as the correct target [12]. The F1 measure is an evaluation that combines both sensitivity (recall) and precision. The calculation formula of the F1 score is as follows:

$$ \mathrm{F}1\ \mathrm{score}=2\times \frac{1}{\frac{1}{Precision}+\frac{1}{Recall}}=2\times \frac{Precision\times Recall}{Precision+ Recall} $$

**Table 2:** YOLO v2 experimental results

Image type	Experiment 1 (Front-side)	Experiment 2 (Back-side)
Training time	5 h 34 mins	7 h 42 mins
Epochs	60	65
Precision	94.09%	96.26%
Recall	94.44%	96.63%
F1 score	93.72%	95.99%

At the same time, we recorded the training time of the model, the number of Epochs in the training, and the classification performance of the model for the testing dataset.

## Results

In this study, two deep learning models were employed for training, and the identification results were compared: the front-side (pill shape and color) model and the back-side (textual pattern and logo) model of blister-packaged drugs. The total training time of the front-side model and the back-side model was 5 h 34 min and 7 h 42 min, respectively. The number of Epochs of the front-side model and the back-side model was 60 and 65, respectively. The precision and recall of the back-side model (96.26 and 96.63%, respectively) were better than those of the front-side model (94.09 and 94.44%, respectively), meaning that texture and logo carried more distinguishing features than were contained in pill shape and color. The F1 score of the back-side model (95.99%) was better than that of the front-side model (93.72%) (Table 2), meaning that when only one model can be used, the back-side model is the preferred choice.

**Fig. 3:**
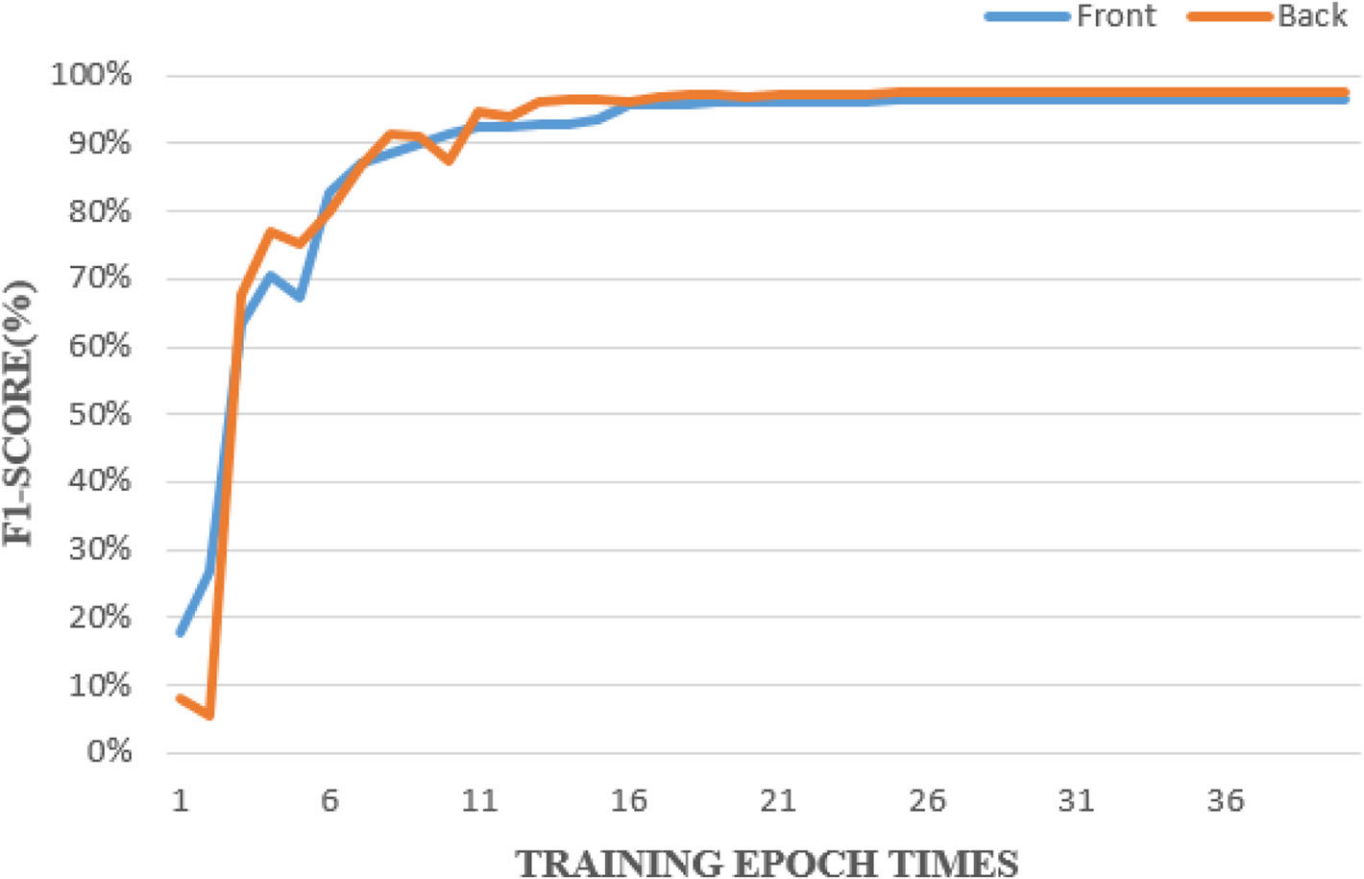
YOLO v2 testing line chart. The F1 score and the correctness rate of identification increased as the number of training Epochs increased; a plateau was then reached when the Epoch number was larger than 8–10, irrespective of front-side or back-side model

In order to show that the identification performance of the deep learning network for blister-package identification is comparable to that of the human eye, we used the YOLO v2 testing line chart to illustrate the results for the front-side and back-side images (Fig. 3). We found that the F1 score increased and the correct rate of identification increased as the training Epoch number increased; a plateau was then reached when the Epoch number was larger than 8–10, irrespective of front-side or back-side model.

Deep learning models share cognitive capabilities similar to those of the human eye, and what confuses a deep learning network can also confuse the human eye. As such, in order to identify look-alike blister packages, we created confusion matrixes, which recorded the actual blister packages that were identified, correctly or not. Correct matches were listed on the diagonal of the matrix, whereas cases of missed identification were marked by non-zero values off the diagonal. The higher the number, the greater the chance of misidentification of blister packages of drugs.

For example, assume that there is a system for classifying three different drugs (Table 3). Suppose that there are 28 drugs in total: 9 drug A, 6 drug B, and 13 drug C. In this confusion matrix, there are actually nine drug A, but three of them are misidentified as drug B. For drug B, one of the drugs is misidentified as drug C, and two are misidentified as drug A. The confusion matrix shows that it is more difficult to distinguish between drug A and drug B, but easier to distinguish drug C from the other drugs. In the confusion matrix, correct identifications are on the diagonal; in contrast, misidentified ones are the non-zero terms off the diagonal.

**Table 3:** Three drugs as an example of a confusion matrix

Predicted class	Drug A	Drug B	Drug C
Actual class			
Drug A	6	3	0
Drug B	2	3	1
Drug C	0	1	12

**Fig. 4:**
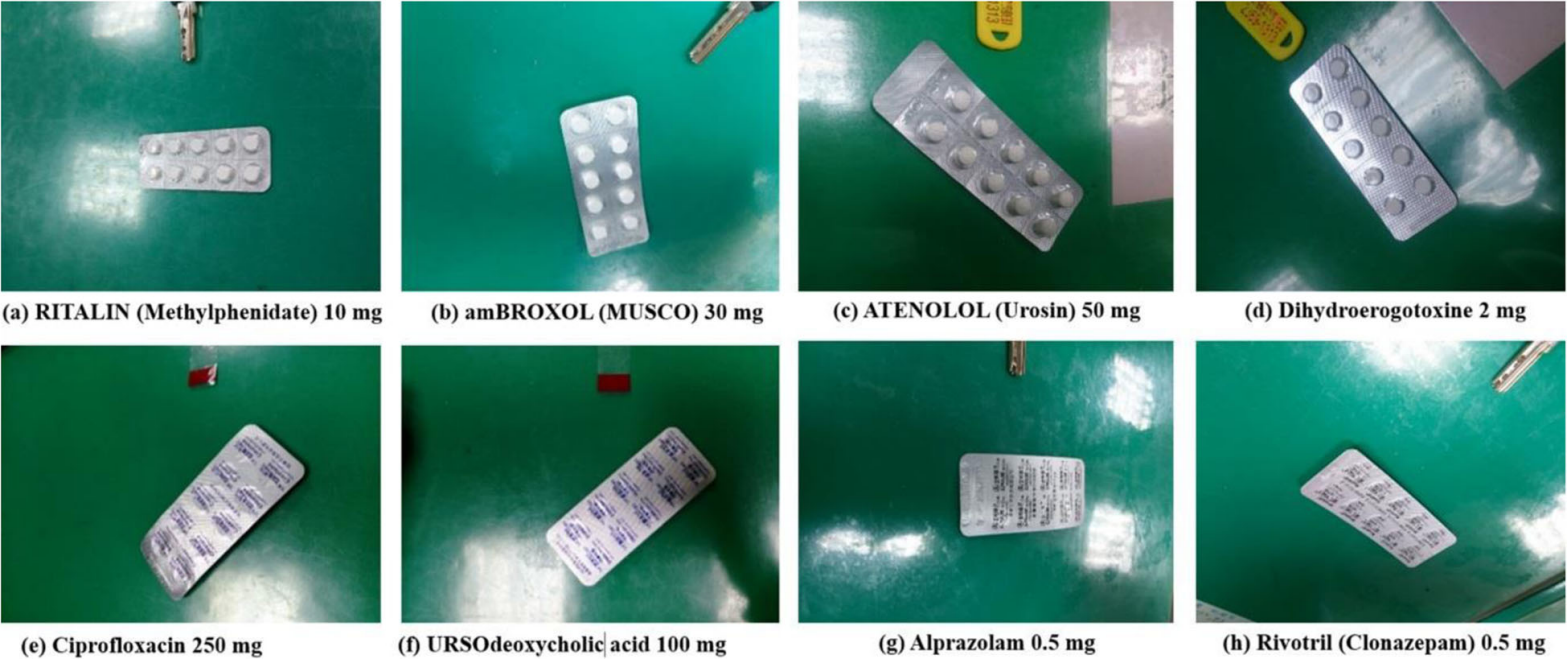
Front and back images of blister packages that were misidentified using the models. The identification results were recorded in confusion matrixes for each model. RITALIN (METHYLPHENIDATE) (**a**) was misidentified as amBROXOL (MUSCO) (**b**) and ATENOLOL (UROSIN) (**c**) was misidentified as DIHYDROEROGOTOXINE (**d**) in the front-side model, while Ciprofloxacin (**e**) was misidentified as URSOdeoxycholic acid (Fig. 4f) and Alprazolam (**g**) was misidentified as Rivotril (CLONAZEPAM) (**h**) in the back-side model.

This study identified two groups of misidentified images based on the confusion matrixes of the two experiments for the front-side and back-side models. According to the identification results recorded in the confusion matrix for the front-side model, the drug RITALIN (METHYLPHENIDATE) (Fig. 4a) has a blister package that was misidentified as amBROXOL (MUSCO) (Fig. 4b). In addition, the blister package of ATENOLOL (UROSIN) (Fig. 4c) was misidentified as DIHYDROEROGOTOXINE (Fig. 4d). This is because the pills shown on the front of the blister packages share the same color and shape, leading to misidentification.

According to the identification results recorded in the confusion matrix for the back-side model, the blister package of Ciprofloxacin (Fig.4e) was misidentified as URSOdeoxycholic acid (Fig. 4f), and the blister package of Alprazolam (Fig. 4g) was misidentified as Rivotril (CLONAZEPAM) (Fig. 4h). These two misidentifications were due to the fact that the backs of the blister packages were wrapped in aluminum foil, and the textual patterns on the back-side were of the same color, without significant difference.

## Discussion

The study provides a qualitative examination regarding how look-alike blister packages are recognized or constrained by deep learning networks that are reminiscent of human visionary cognition capability. With racing speed of progress in deep learning techniques, it is expected that more accurate deep learning solutions will emerge to distinguish nuance image features among different object types, thus mitigating if not solving the dispensing error caused by look-alike blister packages.

Image based techniques, being non-intrusive and without resort to additional devices like RFID tag or bar code, have been a preferred solution to object identification problems. Traditional image-based solutions by computer vision rely on well-defined hierarchical features for effective comparison [34, 35]. Some of the research work from literature reported performance of less than 80% of accuracy with limited number of types of less than 50 [15, 36]. In contrast, the distinguishing features reported in this study are learned by adjusting network parameters through fitting training data, the process being much similar to human visionary recognition process, thus achieving accuracy better than 90% among 250 types. With the advent of deep learning technique, identification witnesses a revolutionary shift which can benefit blister package identification critical to dispensing safety.

This study proposed a novel deep learning drug identification (DLDI) model that delivered satisfactory results for drug identification based on images of blister packages. The results of this study showed that identification by “deep learning” is no less accurate than identification by the human eye. The CNN simulates the response of neurons in the human brain to signals by performing various mathematical operations on features to complete the classification. Repetition of these processes achieves the purpose of recognition. In earlier studies, features were defined subjectively to identify blister packages of drugs [17]. Deep learning allows learning of features automatically, without the need to define features of drugs before machine learning. This advantage eliminates human error and assists pharmacists to identify drugs correctly. Deep learning enables identification of the characteristics of individual drugs clearly and recognizes the drugs that pharmacists/humans consider look alike. Just one or two cameras in dispensing cabinets are required, and medication errors will be prevented.

Referring to Table 2, this study found that back-side images of blister packages of drugs were better than front-side images for identification purposes. While back-side took more training time to better distinguish textual features, based on 4500 test images evenly distributed over 250 types, the associated performance criteria of: precision, recall, and F1 score are all better than that by the front-side images. This is because the information on the back of the packages includes the pharmaceutical company, drug name, dose, and logo in larger text than on the front of the package, which only presents information regarding the color and shape of the pills. The front of the drug packaging contains some three-dimensional information with regards to drug shape. However, some blister packages were not easily recognized by the deep learning network, and were more likely to be confused according to the confusion matrix. These unrecognizable blister packages correspond to look-alike blister packages recognized by the human eye. In the future, we will employ a convolution kernel to identify data features to generate signals and perform a comparison with the human eye.

There are many kinds of drug packages that need to be identified: pills; blister packaging; clip chain bags; powder bags; foil packaging bags; transparent bags; paper packages; bottle packaging, etc. For medication adherence and drug preservation, most drugs are packaged in blisters [37]. Moreover, for some drugs, infrared spectrum analysis of tablets in intact blisters is performed to distinguish between genuine and counterfeit samples [38]. DLDI models may also be applied to automated dispensing cabinets (ADCs), and can be employed in cooperation with both pharmacists and robots. Some robots have cameras, which would be useful for application of our model for drug identification. In the future, we will construct a blister-package identification model that takes account of both sides of the packaging, which will contain more information than just a single side for identification. The identification accuracy may also be increased by use of three-dimensional images of drugs or images with different spectrums for deep learning.

There are some considerations for future studies. First, this study only examined blister-packaged drugs, and used the whole of the blister packages for identification. This model cannot be used to identify blister packages when held in the hand, or trimmed blister packages. Moreover, other types of drug packaging need to be studied. In some cases, the pill size and shape were too familiar to identify. One of the aims of future study is to address these issues. Second, the training time was too long, with more than 5 h required for training the models in this study. More time is required if more than one kind of spectrum is used, and a more effective program is needed to train the models. Third, re-training would be needed if one or more new drugs are added in this model. In the future, we hope to develop a system in which only “PARTIAL” training is required when drugs are changed or added.

## Conclusion

Our goal was to illustrate how ‘look-alike’ error can be captured and explained by a convolution-based deep learning network whose working mechanism is in much similarity to the human visionary recognition capability. Subsequently, appropriate solution to extract more detailed nuance differences can be utilized in distinguishing look-alike objects. With an accuracy greater than 90%, the results of this study may be applied to the real environment, and may assist pharmacists to identify drugs and prevent medication errors caused by look-alike blister packages. The results of this study can also form the core software for robots, allowing filling of prescriptions automatically and preventing medication errors.

## Data Availability

The datasets generated and analyzed during the current study are not publicly available due to that was belonged to the MacKay Memorial Hospital but are available from the corresponding author on reasonable request.

## Abbreviations

ADCs: Automated dispensing cabinets
CNN: Convolution Neural Network
DCN: Deep Convolutional Network
DLDI: Deep learning drug identification
GPUs: Graphics Processing Units
LASA: Look-alike and sound-alike
MLBP: Multi-scale Local Binary Pattern
OPD: Out-Patient Department
RFID: Radio-frequency identification
SIFT: Scale Invariant Feature Transform
YOLO: You Only Look Once
